# Indoor/Outdoor Particulate Matter and Related Pollutants in a Sensitive Public Building in Madrid (Spain)

**DOI:** 10.3390/ijerph22081175

**Published:** 2025-07-25

**Authors:** Elisabeth Alonso-Blanco, Francisco Javier Gómez-Moreno, Elías Díaz-Ramiro, Javier Fernández, Esther Coz, Carlos Yagüe, Carlos Román-Cascón, Dulcenombre Gómez-Garre, Adolfo Narros, Rafael Borge, Begoña Artíñano

**Affiliations:** 1 Department of Environment, Center for Energy, Environmental and Technological Research (CIEMAT), Avenida Complutense 40, 28040 Madrid, Spain; fj.gomez@ciemat.es (F.J.G.-M.); elias.diaz@ciemat.es (E.D.-R.); javier.fernandezg@ciemat.es (J.F.); esther.coz@ciemat.es (E.C.); b.artinano@ciemat.es (B.A.); 2 Departamento de Física de la Tierra y Astrofísica, Universidad Complutense de Madrid, 28040 Madrid, Spain; carlos@ucm.es; 3 Department of Applied Physics, Marine and Environmental Sciences Faculty, INMAR, CEIMAR, University of Cadiz, Puerto Real, 11510 Cádiz, Spain; carlos.roman@uca.es; 4 Laboratorio de Biología Vascular y Microbiota, Hospital Clínico San Carlos, Departamento de Fisiología, Facultad de Medicina, Universidad Complutense de Madrid (UCM), IdISSC, 2nd Floor North, C/Profesor Martín Lagos, s/n, 28040 Madrid, Spain; mgomezgarre@salud.madrid.org; 5 Departamento de Ingeniería Química Industrial y del Medio Ambiente, Universidad Politécnica de Madrid (UPM), 28006 Madrid, Spain; adolfo.narros@upm.es (A.N.); rafael.borge@upm.es (R.B.)

**Keywords:** indoor air quality, indoor/outdoor ratio, hospital, black carbon, ultrafine particle number concentration, particulate matter

## Abstract

According to the World Health Organization (WHO), indoor air quality (IAQ) is becoming a serious global concern due to its significant impact on human health. However, not all relevant health parameters are currently regulated. For example, particle number concentration (PNC) and its associated carbonaceous species, such as black carbon (BC), which are classified as carcinogenic by the International Agency for Research on Cancer (IARC), are not currently regulated. Compared with IAQ studies in other types of buildings, studies focusing on IAQ in hospitals or other healthcare facilities are scarce. Therefore, this study aims to evaluate the impact of these outdoor pollutants, among others, on the indoor environment of a hospital under different atmospheric conditions. To identify the seasonal influence, two different periods of two consecutive seasons (summer 2020 and winter 2021) were selected for the measurements. Regulated pollutants (NO, NO_2_, O_3_, PM_10_, and PM_2.5_) and nonregulated pollutants (PM_1_, PNC, and equivalent BC (eBC)) in outdoor air were simultaneously measured indoor and outdoor. This study also investigated the impact of indoor activities on indoor air quality. In the absence of indoor activities, outdoor sources significantly contribute to indoor traffic-related pollutants. Indoor and outdoor (I-O) measurements showed similar behavior, but indoor concentrations were lower, with peak levels delayed by up to two hours. Seasonal variations in indoor/outdoor (I/O) ratios were lower for particles than for associated gaseous pollutants. Particle infiltration depended on particle size, with it being higher the smaller the particle size. Indoor activities also significantly affected indoor pollutants. PM_x_ (especially PM_10_ and PM_2.5_) concentrations were mainly modulated by walking-induced particle resuspension. Vertical eBC profiles indicated a relatively well-mixed environment. Ventilation through open windows rapidly altered indoor air quality. Outdoor-dominant pollutants (PNC, eBC, and NO_X_) had I/O ratios ≥ 1. Staying in the room with an open window had a synergistic effect, increasing the I/O ratios for all pollutants. Higher I/O ratios were associated with turbulent outdoor conditions in both unoccupied and occupied conditions. Statistically significant differences were observed between stable (TKE ≤ 1 m^2^ s^−2^) and unstable (TKE > 1 m^2^ s^−2^) conditions, except for NO_2_ in summer. This finding was particularly significant when the wind direction was westerly or easterly during unstable conditions. The results of this study highlight the importance of understanding the behavior of indoor particulate matter and related pollutants. These pollutants are highly variable, and knowledge about them is crucial for determining their health effects, particularly in public buildings such as hospitals, where information on IAQ is often limited. More measurement data is particularly important for further research into I-O transport mechanisms, which are essential for developing preventive measures and improving IAQ.

## 1. Introduction

People spend more than 90% of their lives indoors [1]. Indoor pollutants are not as easily dispersed or diluted as outdoor ones, a fact that has led to indoor air quality (IAQ) receiving particular attention in recent years [2,3].

A variety of pollutants and chemicals have been identified in indoor air, originating from indoor sources (e.g., furniture, cleaning products, cooking, smoking, domestic activities or candle use, or human movement) [4,5] but also largely from outdoor sources [6,7], driven by meteorology and atmospheric turbulence [4], which are also modified by building characteristics [8,9]. Outdoor pollutants are the result of natural processes (e.g., atmospheric nucleation) and human activities (e.g., vehicle exhaust or domestic combustion), which release a significant amount of particulate and gaseous pollutants into the atmosphere. However, only a few variables and pollutants are regulated, especially conventional ones such as PM_x_ and NO_x_ [4,10]. Thus, most previous studies investigating the relationship between indoor and outdoor pollutant concentrations have focused on these regulated air pollutants. This is not the case for unregulated air pollutants, such as suspended particles and black carbon (BC), one of their largest constituents.

Suspended particles (aerosols) in the air are recognized as one of the most important pollutants with a potential risk to human health [11]. Due to their small size, aerosol particles can easily enter the respiratory tract and enter the bloodstream during gas exchange, reaching different organs [12]. In addition, the surface-to-volume ratio is inversely proportional to particle size, resulting in a much larger surface area for toxic chemical transfer [13]. Consequently, numerous studies have associated exposure to particulate matter with health problems, including immune, respiratory, cardiovascular, and cerebrovascular diseases [14,15,16].

A key assumption underlying the variables limited by pollutant regulations is their effect on human health. Aerosol particles are typically regulated based on their mass concentration in international and national programs [17,18]. However, this is not a strong enough indicator to assess health risks because it does not include a wide range of variables such as particle number concentration (PNC) or BC particles [13,19,20]. In this respect, the World Health Organization (WHO) distinguishes between low (<1000 particles cm^−3^, 24 h mean) and high (>10,000 particles cm^−3^, 24 h mean or 20,000 particles cm^−3^, 1 h mean) PNC for particles larger than 10 nm [21]. Europe’s new ambient air quality directive [10] is closer to the WHO’s air quality guidelines. For the first time, it includes measuring these pollutants to determine their levels and include them in epidemiological studies. These variables, whose limits are not currently regulated, are reported by the WHO to be universal vectors of a wide variety of toxic chemicals to humans. Their presence, even at relatively low concentrations, can cause adverse health effects [21]. Thus, there is a growing and emerging public concern about these pollutants. This explains the interest in monitoring parameters such as the concentration of ultrafine particles and BC, given that knowledge of the effects of exposure to these unregulated ambient air pollutants is scarcer than that for any other regulated pollutant [20].

Currently, the processes that determine indoor pollutant concentrations are not well understood due to the lack of regulation for a significant number of pollutants that are widely present in both indoor and outdoor environments. These processes are very difficult to identify, and consequently, their impact on indoor air quality is challenging to evaluate. This is due to their being influenced by the immediate outdoor (pollutants and meteorology) environment, the structural characteristics of buildings (e.g., building tightness, type of ventilation, surroundings…), and indoor sources [22] and significant differences in occupant activities (window opening/closing times, walking, cleaning…) [23]. As a result, changes in qualitative and quantitative indoor pollutants can occur within a short period of time. In this context, the modeling of indoor air pollution can be a useful alternative approach [24,25]. However, real pollutant diffusion conditions are more complicated than simulated ones, leading to unexpected discrepancies [26]. Offline analysis does not provide concentration–time profiles of pollutants, which are key to identifying their sources and variations in a given indoor environment. Thus, real-time measurements and their correlation with meteorology can be useful tools for assessing and investigating I/O ratios at high temporal resolution, helping to interpret rapid changes over time due to indoor activities, sources, and sinks [27,28].

As part of the AIRTEC-CM project (Urban Air Quality and Climate Change Integral Assessment: https://airtec-cm.es/), several urban field campaigns were carried out in Madrid to evaluate air quality. These campaigns examined both biotic (pollen and bacteria) [29] and non-biotic (particulate matter and related pollutants) [30] indoor pollutants. Among these campaigns, two consecutive ones were conducted in a hospital building located in the northwest of Madrid in summer (23 June to 11 July 2020) and winter (9–28 February 2021), covering two representative periods in terms of meteorology [31]. The summer data were collected outside the COVID-19 closure period, after all restrictions had been lifted. Therefore, pandemic-associated conditions did not affect the measurements.

Hospitals and healthcare centers are very sensitive and complex environments, inhabited by very fragile individuals, where biotic and abiotic pollutants converge [32,33]. Studies conducted in this type of microenvironment have mainly focused on biotic pollutants such as viruses, bacteria, and fungi and the influence of comfort-related parameters on their concentrations [34,35,36,37,38]. However, far fewer studies have addressed their abiotic indoor air quality [39,40,41,42,43,44]. Therefore, in this context, the present study analyzed the abiotic factors in a hospital’s indoor environment based on three objectives: (1) to assess the impact of outdoor pollutants, particularly particulate matter and related pollutants, on a hospital’s indoor environment under different atmospheric conditions, (2) to compare the I-O concentrations during indoor activities, and (3) to investigate the vertical distribution of outdoor eBC on-site at different building heights. To this end, regulated outdoor pollutants, including PM_10_ and PM_2.5_ fractions, nitrogen oxides (NO, NO_2_, and NO_x_), and ozone (O_3_), as well as the unregulated PM_1_ fraction, PNC, and eBC, were our target compounds. Indoor measurements were performed under a variety of experimental situations and atmospheric conditions. In addition, simultaneous outdoor measurements were recorded to obtain information on outdoor pollution and meteorology. Among them, the vertical distribution of eBC within the height of 100 m above ground level (m a.g.l.) was measured by a drone.

Interestingly, this paper reports on unregulated pollutants in a sensitive building such as a hospital, where IAQ studies are limited. Additionally, the hospital is located in a heavily trafficked area, with high emissions of aerosol particles and gases, which are pollutants that pose a high risk of adverse health effects due to exposure to them.

## 2. Methodology

### 2.1. Air Quality Monitoring Plan and Measurements

The city of Madrid (40° 24′ 59.4” N and 3° 42′ 9.22” W, 657 m above sea level) is the capital of Spain. It is located in the center of the Iberian Peninsula. With over 3 million inhabitants in the city and 7.3 million in the metropolitan area, it is the most populous city in Spain and the center of national economic activities. This results in a high traffic density in the city. The city’s industrial activity consists of some light industry. Consequently, urban pollution levels (e.g., particulate matter, nitrogen oxides, or ozone) are mainly caused by traffic and domestic activities, particularly during the cold months [45], when meteorological conditions are more stable. Regarding particulate air pollution, atmospheric nucleation processes mainly contribute to the presence of ultrafine particles during the warm months [46]. In addition, Madrid is also frequently affected by Saharan dust outbreaks in the spring and summer, and less frequently in the autumn, which contribute to increased PM_s_ concentrations in the city [47].

The region of Madrid has a continental Mediterranean climate, which is tempered by the urban heat island effect [48]. In addition, due to its proximity to the Guadarrama mountain range (~40 km northwest of Madrid), the city’s winds are dominated by mountain breezes, which can significantly affect pollutant concentrations [49,50].

**Figure 1:**
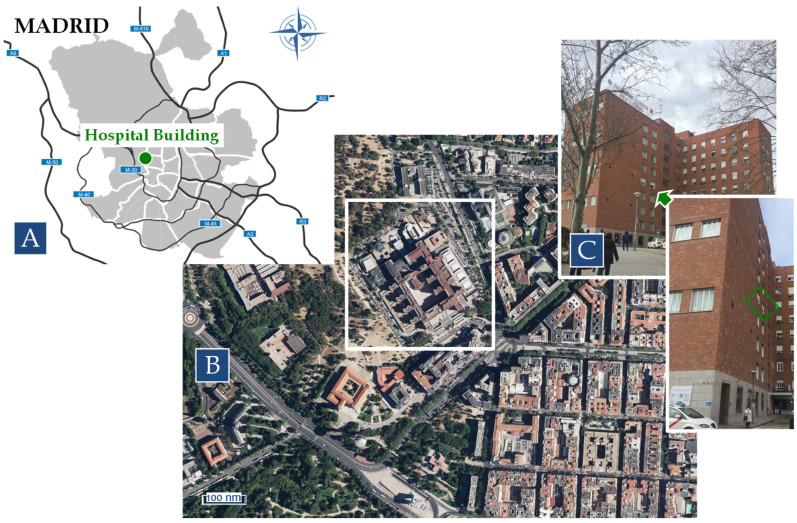
Location of hospital building in Madrid (**A**), surrounding area (**B**), and sampling point (**C**). The green arrow indicates the floor of the building where the measuring equipment was installed. The green frame indicates the location of the windows from which the outdoor measurements were taken.

Outdoor and indoor pollutants were measured simultaneously in a hospital (40°26′26′′ N 3°43′13′′ W) during two intensive observation periods, covering two consecutive and representative periods of summer (June–July 2020) and winter (February 2021). The location of the measurement points is shown in Figure 1. The hospital is located northwest of the city of Madrid, ~3 km from the city center. It is situated in an open square with high urban traffic density and is surrounded by busy streets (Figure 1). In addition to traffic emissions from the surrounding streets, emissions from ambulances, garbage trucks, and hospital vehicles contribute significantly to local air pollution outside the hospital building. No indoor sources were detected.

The indoor sampling point was located in a multi-purpose room situated on the fourth floor of an 8-story hospital. The room had only one door and three east-facing windows. The outdoor measuring instruments were installed inside the room. The sampling line was set at a horizontal level of ~20 m above ground level (a.g.l.) and extended outside through a room window ~2 m from the wall. The window was then well-sealed to prevent drafts and air leakage. The location of the equipment and the length of the lines were optimized to avoid uncertainties in the measurement response. A lack of hospital authorization prevented the exploration of indoor air quality in other areas of the hospital, such as waiting and inpatient rooms.

Ultrafine particle number concentration (PNC) was monitored using condensation particle counters (CPCs: TSI-3772 and MAGIC™ models). Particle mass concentrations (PM_10_, PM_2.5_, and PM_1_) were measured using Grimm EDM 365 and 11D systems. Exceptionally, outdoor measurements during the summer campaigns were completed with a TEOM^®^ 1405-DF installed at ground level at the CIEMAT suburban site, ~2 km from the hospital building [51], due to technical problems, with the Grimm model 11D used during the winter campaign. Portable microAeth^®^ AE51 and MA200 models were used to optically measure BC, referred to as mass-equivalent BC (eBC) concentrations [52]. BC is analyzed by collecting particles on a replaceable filter strip and determining their mass concentration in real time using the Aethalometer^®^ optical absorption technique. This type of instrument has been used by the research group in previous indoor and outdoor air quality studies [53,54,55].

In addition, vertical concentration profiles of eBC were obtained using a drone equipped with a microAeth^®^ model AE51. Data were collected at different heights (0, 5, 10, 15, 20 (height of the outdoor sampling point), 30, 50, 60, 80, and 100 m a.g.l.) with an average flight time per profile of ~1 h. NO, NO_2_, and O_3_ concentrations were measured simultaneously using 42i and 42i-TL NO_x_ analyzers and a Sabio model 6030 O_3_ analyzer, respectively. No ozone data were available during the summer campaign due to a lack of instrumentation. For NO_2_, indoor and outdoor measurements were also sampled with Palmes-type passive diffusion tubes (PDTs) for weekly periods, with a total of 4 tubes (2 indoor and 2 outdoor) in each campaign. These sampling periods were considered when studying the I/O ratios, which were defined as periods 1 and 2 in each campaign (see Section 3.2). All instruments were calibrated and/or intercompared prior to the campaigns. The CPCs were compared during the annual REDMAAS intercomparisons [56] and with the SMPS at the Madrid station, which is part of the European ACTRIS infrastructure (https://www.actris.eu/ (accessed on 23 April 2025)). The portable microAeth^®^ AE51 was also compared with a continuous aethalometer at the Madrid station, which is also part of ACTRIS. The Grimms were compared with the TEOM, which is calibrated annually by the manufacturer. Finally, the gas analyzers (NO, NO_2_, and O_3_) were calibrated in the laboratory and then compared. Measurements from the different devices were consistent with each other and remained within the established uncertainty range of the manufacturers.

Table 1 lists the characteristics of the instrumentation used in each campaign, and Table S1 shows the data coverage for the different measured outdoor and indoor pollutant parameters.

Moreover, meteorological (ambient temperature (T), relative humidity (RH), wind speed (WS), and wind direction (WD)) and micrometeorological (Turbulent Kinetic Energy (TKE), friction velocity (u*), and Sensible Heat flux (SH)) parameters were measured and calculated to obtain the local meteorology and turbulent conditions. For this purpose, a portable meteorological station equipped with an eddy covariance system was installed on the roof of the hospital building (~34 m a.g.l.). Measurements were taken continuously and averaged over 10 min. The meteorological information, especially the turbulence variables, was used as a predictor to correctly explain the temporal variability in indoor and outdoor pollutants caused by small-scale atmospheric processes. More detailed information on the meteorological aspects of the hospital building can be found in Román-Cascón et al. [50], where the same portable station was used for other field campaigns in Madrid. Additionally, this work considered reference regional and local meteorological information provided by the CIEMAT instrumented meteorological tower [51], including wind speed and direction, temperature at two different heights, relative humidity, atmospheric pressure, and solar radiation. The CIEMAT tower is located ~2 km from the hospital building and is surrounded by natural areas on three sides (southwest, northwest, and northeast). It is not affected by local buildings or other tall structures, making it representative of the general atmospheric situation in the urban area.

All parameters, both ambient air pollutants and meteorological parameters, were monitored in real time with time resolutions between 1 s and 10 min. For comparison purposes, the data were averaged at the lowest resolution measured in this study, 10 min. The data presented here are in UTC time.

In order to identify the origin of the air masses over Madrid, HYSPLIT (Hybrid Single-Particle Lagrangian Integrated Trajectory, https://www.ready.noaa.gov/HYSPLIT.php (accessed on 19 November 2024)) simulations at 500, 1500, and 3000 m a.g.l. were conducted for all the days with runtimes of −72 h ending at 12:00 UTC. Furthermore, the Dust Regional Atmospheric Modeling (DREAM) system (http://www.bsc.es/ (accessed on 23 October 2024)) was used to detect the presence of Saharan dust outbreaks over Madrid during the measurement campaigns. Additional synoptic maps provided by the Spanish Meteorological Agency (https://www.aemet.es/ (accessed on 21 November 2024)) were used to interpret the atmospheric meteorological situation.

All activities in the hospital room were recorded in a time–activity diary. Measurements were obtained under different scenarios including unoccupied (with the door and windows kept closed and no one in the room), occupied (with the operator(s) present to check the instruments), with the window open, and with the window open + occupied. Conventional activities in the multi-purpose room were stopped for the development of the campaign, which allowed us to characterize the effects of outdoor pollutants infiltrating the indoor environment, as well as the effects of different indoor activities on indoor air quality. Unoccupied periods accounted for 81% of the summer campaign and 73% of the winter campaign. The data distribution in relation to the activity period in the room is as follows: 1, 6, and 0.5% in the summer and 2, 7, and 1% in the winter for occupancy, open window, and occupancy + open window conditions, respectively. No other indoor activities besides those described in this study were carried out in the room.

### 2.2. Data Analysis

In the absence of indoor activity, without indoor pollutant emissions, and under steady-state indoor conditions, the infiltration factor (*F*_*in*_) can be formulated as the I/O ratio [57]. Thus, this indicator is the most commonly used to characterize the contribution of outdoor pollutants to indoor environments [58]. The premises established by Long et al. [57] are consistent with those of this study, so the I/O ratio is also used to estimate the infiltration factor here.

The I/O ratios for the different scenarios were evaluated separately. Since the ratios follow a lognormal distribution, the main descriptive statistic used in this study was the geometric mean. An I/O ratio ≤ 1 indicates that indoor pollutants did not reach outdoor levels, while a ratio greater than 1 indicates that indoor pollutants reached or exceeded outdoor levels. The 95% confidence intervals around the estimated geometric mean were calculated. If the value of 1 is within the confidence interval, the results are inconclusive, and it cannot be determined whether the highest concentration is indoor or outdoor.

A statistical analysis was performed using the R software tool to further analyze the results obtained from the measurement campaigns. Student’s t-test *p*-values were calculated to determine the differences between the hourly mean I/O values obtained for the two measurement periods and the influence of atmospheric stability, using the TKE parameter as an indicator of the atmospheric diffusion (values of 1 ≤ m^2^ s^−2^ indicate stable conditions, while values of 1 > m^2^ s^−2^ indicate unstable conditions). Subsequently, a linear regression model was used to explore the relationship between the I/O pollutant ratios and the wind direction factor. This analysis took into account the eight directional sectors (N, NE, E, SE, S, SW, W, and NW) during periods of atmospheric instability (TKE > 1 m^2^ s^−2^) for the summer and winter campaigns. The significance level was set at 0.05.

Information on air exchange rates and the area of the open window is unavailable. The room was ventilated through a window in the center, which always allowed for the same air exchange area. Studying the I/O ratios in relation to Turbulent Kinetic Energy (TKE) provides indirect information on this issue. Since the open window was the only ventilation system in the room, atmospheric turbulence conditions determined the entry of indoor pollutants and indirectly indicated the air exchange rates.

## 3. Results and Discussion

### 3.1. Meteorological Aspects and Air Pollution

The data sets represent two opposite seasonal meteorological conditions (summer vs. winter), during which the outdoor pollutant concentrations in Madrid differ significantly. Therefore, knowledge of the air mass trajectories and meteorological conditions (stability/turbulence) is essential to correctly interpret the indoor/outdoor pollutant measurements obtained during the field campaigns.

Composite anomaly fields of the 500 hPa geopotential height (Z500) and sea level pressure (MSLP) showed that the summer campaign was more stable compared than normal summer climatology, while the winter campaign was more unstable than the climatological values.

**Figure 2:**
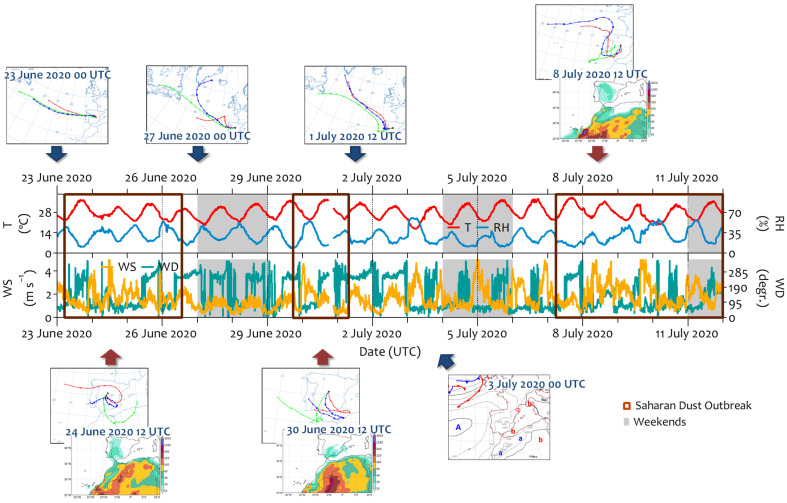
The temporal evolution of meteorological information (temperature (T), relative humidity (RH), and wind speed and direction (WS and WD, respectively)) recorded at the meteorological station located on the roof of the hospital building during the field campaign in June–July 2020. The main back trajectories, the sea level pressure, and dust maps describing the different atmospheric situations identified in this study are included in this figure.

No precipitation was recorded during the summer, and temperatures were high, ranging from 16.6 to 38.1 °C (Figure 2). Three Saharan dust outbreaks (23–26 June 2020, 29 June–1 July 2020, and 7–11 July 2020) were identified, which are relatively typical phenomena over the Iberian Peninsula. These episodes had a strong impact on ground-level PM_x_. Although the daily PM_10_ limit (50 µg m^−3^) concentration was not exceeded, the hourly concentration reached 91 µg m^−3^ (8 July 2020, 17:00 UTC). PM_2.5_ increased significantly as well, with hourly maximums exceeding 20 μg m^−3^ (8 July 2020). The stable low-level atmosphere consequently inhibited the dispersion of pollutants during these periods, causing them to accumulate on the surface. Thus, traffic-related air pollutant concentrations increased, especially those for PNC (>20,000 particles cm^−3^/h, the hourly value recommended by the WHO), eBC, and NO_x_. Outside these periods, the trajectories of the air masses arriving in Madrid came from the Atlantic regions. On June 27 and 28, the air masses came from the northwest direction with wind speeds of ~2 m s^−1^. However, from July 2, the presence of an anticyclone system over the Iberian Peninsula was associated with shorter trajectories at lower levels (Figure 2). This caused pollutants to accumulate at the surface level until July 7 (Figure S1). In any case, outdoor concentrations in the summer were characterized by lower values than those in the winter, especially for traffic-related pollutants (Figure S2). This is because summer is characterized by intense convective atmospheric dynamics, higher temperatures, increased solar radiation, and reduced local emissions, especially from traffic, compared to winter.

**Figure 3:**
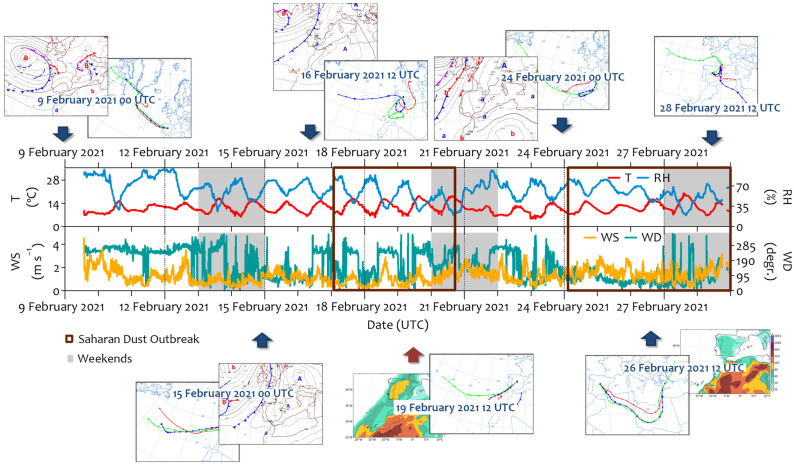
The temporal evolution of meteorological information (temperature (T), relative humidity (RH), and wind speed and direction (WS and WD, respectively)) recorded at the meteorological station located on the roof of the hospital building during the field campaign in February 2021. The main back trajectories, the sea level pressure, and dust maps describing the different atmospheric situations identified in this study are included in this figure.

In contrast, the winter campaign (2021) was a predominantly unstable period. Precipitation was recorded for several days (February 9–12 and 21–22), with an accumulation of ~38.5 mm (measured at the CIEMAT meteorological station, located ~2 km from the site). An analysis of back trajectories during these days showed that the air masses arrived mostly originated from the North Atlantic region (Figure 3). Consequently, the relative humidity reached 100 %, and the temperature did not register negative values (4.6–20.0 °C). These conditions were interrupted by two intense Saharan dust outbreaks (17–21 February and 24–28 2021). The contribution of Saharan dust to PM_10_ and PM_2.5_ was higher than that during the summer campaign (Figure S3). The daily PM_10_ limit (50 µg m^−3^) was exceeded on February 18 (91.4 µg m^−3^), 19 (84.3 µg m^−3^), 21 (57.4 µg m^−3^), and 25 (53.5 µg m^−3^), 2021, with a significant contribution of PM_2.5_ (up to ~50% of PM_10_). Consequently, high atmospheric stability and poor dispersion conditions favored the accumulation of pollutants during these days. Unlike PM_x_, the polar plots indicate that PNC, BC, and NO_x_ have a clear local origin from traffic emissions (Figure S2). Additionally, ultrafine particles and eBC showed pronounced peaks that coincided with traffic emissions. Concentrations higher than 10,000 particles cm^−3^ were usually found outdoor during the winter, with those in peak hours being higher than 20,000 particles cm^−3^, which exceeds the WHO hourly recommendation [21]. The eBC concentration was also higher, with peaks of up to 6.5 µg m^−3^. NO_2_ concentrations showed higher values, while those of ozone decreased due to titration reactions with NO (Figure S3). This inverse relationship reflects typical urban atmospheric chemistry, in which elevated NO emissions (often from traffic or combustion sources) react with ozone, leading to ozone depletion and an increase in NO_2_ levels.

According to the Madrid City Council’s latest air quality report [59], all pollutants decreased slightly in 2020 due to stringent lockdown measures in the first half of the year. However, the summer campaign was carried out during the post-COVID-19 period, when all COVID-19 restrictions were lifted, and the measurements are comparable to those observed during a non-COVID-19 period. Additionally, slight changes in the absolute concentrations of the pollutants do not affect their relative values, which are examined in this study. Thus, the measurements obtained during these campaigns are representative of a warm period (summer) and another cold period (winter) in Madrid.

Despite the restrictions imposed by the COVID-19 pandemic, several air pollutants showed significant improvement (or remained constant) compared to pre-pandemic levels. This improvement was seen in pollutants such as NO_2_, SO_2_, and CO due to the local air quality plans, technological changes in vehicles and heating systems, and other government actions. However, particulate matter (PMₓ), one of the most harmful pollutants to human health, has increased. For example, the annual mean concentrations of PM_10_ recorded by the Madrid City Council’s air quality network increased from 17.5 µg m^−3^ in 2019 to 20.8 µg m^−3^ in 2022. Other natural pollutants, such as ozone (a secondary pollutant), have also increased in recent years.

### 3.2. Indoor-to-Outdoor Ratios Under Different Outdoor (Sources and Meteorology) Conditions

In the absence of indoor sources in the hospital room, outdoor air pollution had an important influence on the indoor environment. Thus, the temporal variation in traffic-related indoor pollutants followed that of outdoor ones. However, the indoor concentrations were usually lower than the outdoor ones (Figures S1–S3) and were higher in winter than in summer. There was a clear diurnal pattern, but it was smoother and showed a slight delay indoors, which is consistent with previous studies [4,60]. A time lag of up to 2 h was found between the maximum indoor and outdoor concentrations of each pollutant. However, an exact estimate was not possible. As other authors have pointed out, there are many factors influencing the time lag, which could be related to the nature of the pollutant (gas or particulate), its reactivity, and pollution-related factors such as emission characteristics and meteorology [61,62]. This explains why a wide range of lag times can be found in the literature. For example, the lag time for PM_2.5_ particles has been reported to range from ~1 h [63] to a few hours [64,65]. These differences may be related to outdoor particle sizes (i.e., their origin), consistent with other studies [26,66]. Reche et al. [67] found a similar lag time estimate of ~1 h for eBC, the aerosol component contained in the fine fraction. Regarding numerical particle concentrations, Miller et al. [60] found weekend nighttime lags of 1 to ~2.5 h for PM_1_ when air conditioning was not in use. For gaseous pollutants, Challoner and Gill [62] found lag times of up to 1 h between indoor and outdoor NO_2_ variations in commercial buildings in Dublin.

I-O concentrations showed linear correlations (R^2^) higher than 0.5 for PM_2.5_ (0.6) in summer 2020 and for PM_10_ (0.7), PM_2.5_ (0.8), PM_1_ (0.5), eBC (0.5), and NO (0.5) in winter 2021. O_3_ concentrations had a relatively low degree of correlation (0.3), as also found by Yang et al. [68]. Although some pollutants are not highly correlated between indoor and outdoor environments, this is due to lag times rather than an absence of indoor–outdoor relationships. On days with significant traffic emissions, peaks in the outdoor concentrations of PNC, eBC, and NOx associated with traffic were reflected in indoor levels. This indicates the infiltration of these outdoor pollutants, as demonstrated by hospital studies [69,70]. However, indoor NO_x_ concentrations were higher than outdoor concentrations, particularly at night, when the reactivity of these gaseous pollutants is low [71] and decreases more slowly than outdoor concentrations [62].

In general, higher I/O ratios (from 0.25 for PM_10_ to 0.95 for NO and NO_2_) were observed during the winter field campaign than during the summer campaign (from 0.17 for PM_10_ to 1.05 for NO_2_) (Table 2). In the summer, although O_3_ data were not available in the room, a reduction in NO emissions and an increase in NO titration as a consequence of a greater O_3_ formation could have increased the NO_2_ concentration. Thus, the NO I/O ratios were lower than those in winter (Table 2). The lowest I/O ratios for gaseous pollutants were recorded for NO in summer and for O_3_ in winter. In the latter case, studies on indoor ozone removal report that occupants (their exposed skin, hair, and clothing) and furniture are significant ozone sinks due to surface interactions [72,73], reducing its indoor concentration for the production of secondary pollutants. The results of monitoring NO_2_ by passive sampling were similar to real-time measurements during non-occupancy periods (Table 2).

**Figure 4:**
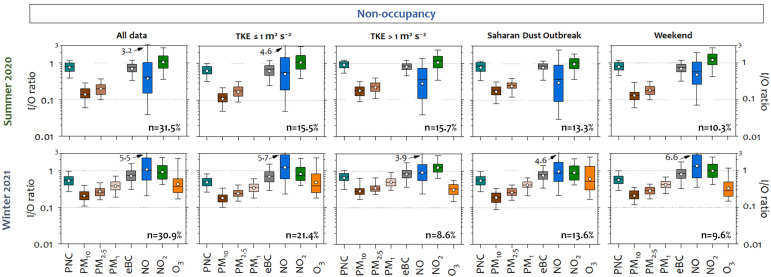
A box plot of the I/O pollutant (PNC, PM_10_, PM_2.5_, PM_1_, eBC, NO, NO_2_, and O_3_) ratios measured in each measurement campaign in the absence of indoor activities, analyzing all data and data obtained during Saharan dust outbreak weekends and under different atmospheric dispersion conditions categorized by the Turbulent Kinetic Energy (TKE) parameter. The box defines the 25th, 50th, and 75th percentiles, while the whiskers define the range from the 5th to 95th percentile. The mean value for each pollutant is plotted as the dot. Each box plot shows the sample size as a percentage.

Local outdoor turbulence conditions seemed to control the indoor/outdoor relationships. Considering the whole period of each campaign and those corresponding to the installation of the passive tubes (periods 1 and 2), an increase in the I/O ratios was observed for traffic-related pollutants in summer during period 2. This could be due to slower indoor response times to outdoor changes when the Turbulent Kinetic Energy (TKE) was higher (Table 2). Based on this finding, the relationship between indoor and outdoor pollutants during the field campaigns was discussed in terms of meteorology, particularly TKE, as well as sources, using the I/O ratios during non-occupied periods. A sensitivity test was performed to evaluate the relationship between the I/O ratios and TKE, with clear differences observed for values higher than 1 m^2^ s^−2^ (Figure S4). Figure 4 shows box–whisker plots of the I/O pollutant ratios under different dispersive (outdoor) conditions.

Infiltration is more efficient as the particle size is smaller, the most harmful to health [74,75]. This fact was clearly observed in the winter campaign. The I/O ratios were the lowest for PM_10_ (0.25), followed by PM_2.5_ (0.30) and PM_1_ (0.42). A similar behavior of the I/O ratios was found for PM_10_ and PM_2.5_ during the summer campaign. This behavior is expected as a consequence of the higher deposition rates caused by gravitational sedimentation or inertial deposition for the largest particles (especially PM_10_), resulting in a lower infiltration factor [76,77]. Studies of indoor air quality in hospitals located in urban areas have also shown significantly higher concentrations of fine particles (<PM_2.5_) than coarse particles in hospitals located in predominantly commercial or industrial areas [69,78]. These findings highlight the critical influence of surrounding environmental sources on indoor air quality in healthcare facilities.

Saharan dust outbreaks did not seem to affect the PM_x_ I/O ratios, but indoor levels were elevated, especially for PM_10_ (up to 42.8 µg m^−3^ vs. 202.5 µg m^−3^ outdoor) and PM_2.5_ (up to 19.1 vs. 70.3 µg m^−3^ outdoor) during the winter campaign. This suggests a direct link with outdoor air quality and the indoor PM levels, as observed in other hospitals [79]. Other studies have found high indoor PM_2.5_ and PM_10_ levels during heat wave days [80] or dust storms [4], depending on their intensity and duration. For PNC, the I/O ratio was higher than that of PM_x_ (0.78 in summer and 0.58 in winter, on average), as this is mainly driven by ultrafine particles (<100 nm) [81,82], and these particles have a higher infiltration capacity [83]. The average outdoor particle concentrations exceeded 10,000 particles cm^−3^ (the hourly value recommended by the WHO) in winter, especially when Turbulent Kinetic Energy (TKE) was ≤1 m^2^ s^−2^. In contrast, indoor particle concentrations due to infiltration did not reach these levels.

eBC is one of the main contributors to the fine aerosol fraction [84,85]. The eBC I/O ratios were similar in both campaigns (~0.7) and higher than those for PNC in winter. Since traffic is the main source of BC in the city of Madrid in both winter and summer [86], a possible explanation for this fact is that the concentration of recent fresh particles, with a smaller size, emitted by vehicles in winter was higher than that in summer [87,88]. Due to the lower removal rate of these particles in indoor environments, eBC resulted in an increase in indoor air, where higher eBC I/O ratios were associated with the presence of smaller particles from combustion processes [89,90]. The WHO has classified this pollutant as a Category I Carcinogen, and it is associated with adverse health effects [91,92].

The greatest variability in I/O ratios was observed for the gaseous pollutants. NO_2_ showed no apparent difference between outdoor and indoor concentrations in summer (Table S2), while the I/O ratios for NO and NO_2_ were about 1 in winter. These gaseous pollutants are more reactive than particulate ones, and thus, they can react with surfaces and other air pollutants once they are in the indoor environment [93,94]. These reactions include the photolysis of NO_2_ outdoors and the back-reaction of NO and O_3_ to NO_2_ indoors, which can result in NO_2_ I/O ratios greater than 1 [4]. Some authors have identified NO_2_ accumulation in the indoor air as a result of poor ventilation to prevent heat loss and an increase in outdoor concentrations in winter [95]. This accumulation has also been identified at nighttime due to low outdoor concentrations while ventilation systems are turned off [62]. In this study, the indoor NO_x_ accumulation could be the result of poor indoor air renewal, particularly at night.

The I/O ratios reported in other studies vary widely. For example, in the absence of indoor sources in different types of buildings, the I/O ratios of traffic-related air pollutant are less than 1 [96,97]. In hospital rooms in Erfurt (Germany), the PM_2.5_ I/O ratio was 0.63 [98]. Regarding particle number concentration, the I/O ratio was 0.33, and the I/O ratio of associated carbonaceous pollutants, such as eBC, was 0.44 [98]. I/O ratios below 1 were also identified for gaseous pollutants. For example, a NO_2_ I/O ratio of 0.13 was found in UK apartments [99], while a lower ratio of 0.03 was found in ten commercial buildings, as reported by Challoner and Gill [62]. The O_3_ I/O ratios varied from 0.2 to 0.7 in Yangon [100] and from 0.21 to 0.39 in student dormitories in Nanjing (China) [68]. However, the presence of multiple indoor sources of PNC or eBC, such as smoking, fireplaces, candles, etc., or potential reactions in the indoor environment may lead to higher I/O ratios [4,76,101].

In the absence of indoor sources, many factors, including meteorology, play a significant role in indoor pollutants. Stability/turbulence (considering TKE) affect I/O ratios. Higher values of traffic-related air pollutants (PNC, eBC, NO, and NO_2_) were found when highly dispersive atmospheric conditions (TKE > 1 m^2^ s^−2^) occurred in both campaigns. Rapid changes in outdoor concentrations lead to higher I/O ratios, altering the air exchange rate, as pointed out by Wan et al. [102], and this has been documented in hospital air quality studies by Mohammadyan et al. [70]. Except for NO_2_ in summer, there are statistically significant differences (*p* < 0.05) in I/O ratios between stable (TKE ≤ 1 m^2^/s^2^) and unstable (TKE > 1 m^2^/s^2^) conditions (Table S3). These results highlight the direct influence of window airtightness and the building envelope on the infiltration capacity, confirming the results shown in Table 2. The influence of wind direction was also analyzed during unstable (TKE > 1 m^2^ s^−2^) conditions using a linear regression model. The model summary is presented in Table S4. Westerly winds, i.e., those opposite to the direction of the window, affected the I/O ratios and decreased them. This could indicate a turbulence effect in the sampling area. Easterly winds, which correspond to traffic roads, also have an important effect on the decrease in I/O ratios, meaning that outdoor concentrations are higher than indoor ones. Other authors have studied the influence of outdoor meteorology on the indoor environment and report conflicting results on the influence of meteorological parameters on IAQ. Associations have been found with temperature due to the indoor–outdoor gradient [96,103], relative humidity, or wind speed [102,104].

Although information on the characteristics of the hospital building is unavailable for this study, several studies have highlighted that the importance of building location is an important factor to consider for indoor air quality (IAQ), because it determines the infiltration of outdoor-generated pollutants through cracks and leaks [105,106].

### 3.3. Indoor Activities and Their Effects on Aerosol Particles and Related Pollutants

**Figure 5:**
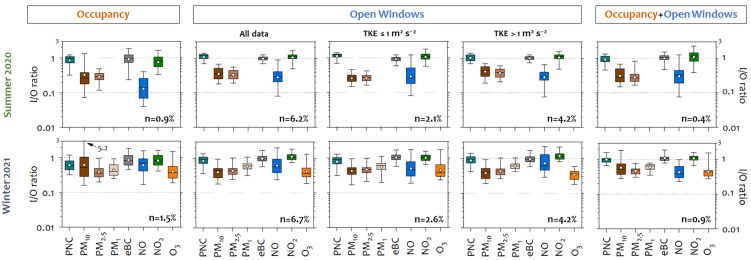
A box plot of the I/O pollutant (PNC, PM_10_, PM_2.5_, PM_1_, eBC, NO, NO_2_, and O_3_) ratios measured in each measurement campaign during indoor activities (occupancy, open windows, and occupancy + open windows) and under different dispersion conditions categorized by the Turbulent Kinetic Energy (TKE) parameter. The box defines the 25th, 50th, and 75th percentiles, while the whiskers define the range from the 5th to 95th percentile. The mean value for each pollutant is plotted as the dot. Each box plot shows the sample size as a percentage.

Ventilation and occupant activities have also been identified as important factors contributing to the indoor levels of these pollutants. Therefore, the impact of these activities on IAQ was evaluated under different dispersive (outdoor) conditions. Figure 5 shows a box plot of the I/O pollutant ratios during indoor activities under different dispersive atmospheric conditions in both field campaigns.

 I/O ratios depended heavily on indoor activities and were susceptible to rapid changes depending on environmental conditions. Occupancy strongly influenced indoor pollutant concentrations. Compared to PM_2.5_ and PM_1_, resuspension was the most significant source of larger particles (PM_10_). These results are consistent with previous studies [107,108], including those conducted in occupied indoor hospital environments [109]. Thatcher and Layton [110] found a 100% increase in the concentration of supermicron particles just by entering a room. Similarly, Serfozo et al. [111] found an average increase of 84% during walking experiments in a laboratory. This study found a remarkable and significant increase in PM_x_, especially when more than two people occupied a hospital room for over 30 min, reaching levels of 42.4 µg m^−3^ (instantaneous I/O ratio of 11.8) for PM_10_. This meant that indoor levels were much higher than outdoor levels. Consequently, the worst correlations between PM_x_ I/O concentrations were found when indoor sources (resuspension) predominated over outdoor (infiltration) ones. Walking activity had no effect on PNC. Indoor pollutants returned to pre-occupancy levels within several minutes to approximately 30 min after occupants left the room.

Natural ventilation through open windows was evaluated under different dispersive atmospheric conditions. Regardless of the outdoor air pollution level, a very rapid increase in traffic-related pollutants (PNC, eBC, and NO_x_) was observed during ventilation. Consequently, ratios close to 1 or higher were found for PNC (1.09 in summer and 0.84 in winter), eBC (0.97 in summer and 0.99 in winter), and NO_2_ (1.06 in summer and 1.12 in winter) in both campaigns, as identified in other studies [83,108]. These ratios were slightly higher under dispersive conditions (TKE > 1 m^2^ s^−^^2^), which was attributed to the fact that the pollutant concentration changes rapidly outdoors and more slowly indoors. However, other authors, such as Zhao et al. [104], Chithra and Nagendra [112], and Elbayoumi et al. [113], found negative correlations with wind speed, i.e., the higher the speed, the lower the particle I/O ratios. In winter, PM_x_ may have a greater impact than in summer with open windows, because higher values are reached during Saharan outbreaks. It can certainly be expected that changes in ventilation will affect pollutant gases differently than particles due to their higher reactivity and diffusion capacity, which increases indoor NO_2_ concentrations (Figure 5). During the open window periods, higher pollutant I/O ratios were also observed when TKE was greater than 1 m^2^ s^−2^, associated with rapid changes in outdoor concentrations. In this regard, indoor particle concentrations exceed 10,000 particles cm^−3^, which is even higher than outdoor ones, especially in the summer.

Indoor/outdoor ratios were lower for shorter ventilation periods than for longer ones. Ventilation rates increased the proportion of outdoor pollutants entering the indoors [114]. Several studies have shown that human behavior indoors affects IAQ, so this information can help reduce occupant exposure to outdoor pollutants indoors [99]. When windows were closed, outdoor air infiltration was restricted, and the relatively high I/O ratios were maintained for hours until stable conditions were restored. In this sense, air-conditioned hospital environments appear to reduce indoor aerosols but have no significant effect on gaseous pollutants [115,116].

There was probably a synergistic effect between occupancy and open windows. Compared to other scenarios, indoor concentrations were increased for all measured pollutants, with the higher concentrations identified for PM_10_ (up to 106.2 µg m^−3^, instantaneous I/O ratio of 0.7) and PM_2.5_ (30.8 µg m^−3^, instantaneous I/O ratio of 0.6). The door was opened only when entering or leaving the hospital room, and no change in indoor concentrations was observed. This indicates that the immediate environment of the room does not affect indoor air quality.

### 3.4. Vertical Profiles of eBC

**Figure 6:**
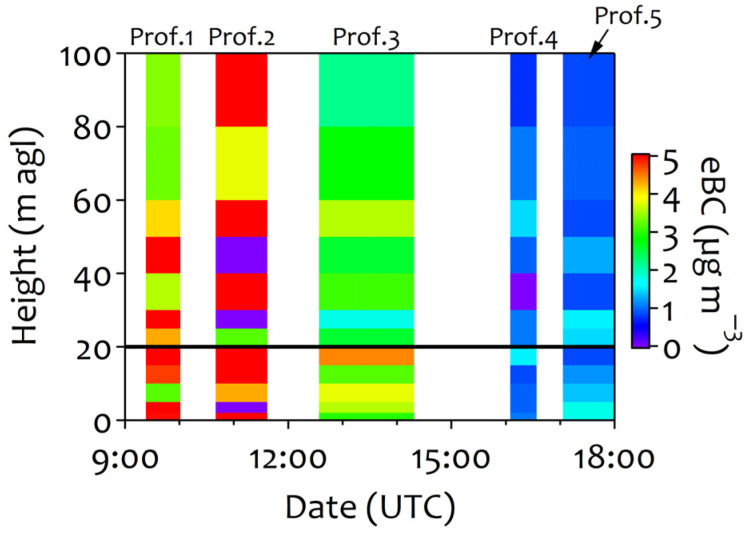
The vertical profiles of eBC measured in the hospital building’s surroundings on 17 February 2021. The indoor/outdoor sampling point was located ~20 m a.g.l.. In the graph, Prof. refers to profiles.

A case study was conducted on 17 February 2021 to investigate the eBC vertical profile (Figure 6). Five complete vertical profiles (from 0 to 100 m a.g.l.) were sampled between 8:54 and 17:30 UTC, lasting between 50 and 77 min. The differences in the times of each profile were due to measurement failures or changes in the drone’s battery. A Saharan dust outbreak occurred during the sampling period. The temperature varied between 9.8 and 17.1 °C with a mean relative humidity of 52%. Poor turbulent conditions were recorded, with an average TKE of 0.6 m^2^ s^−2^ and a south–southwest direction. Due to the high atmospheric stability, air quality in Madrid was poor. PM_10_ and PM_2.5_ concentrations reached 38.0 and 22.0 μg m^−3^, respectively. Traffic-related pollutants were accumulated at the surface, reaching significant concentrations of PNC (1.6 × 10^4^ cm^−3^), eBC (2.1 µg m^−3^), and NO_2_ (33.2 µg m^−3^). At a height of 20 m, where the permanent indoor/outdoor sampling point was located, the mean outdoor eBC concentration was 2.5 µg m^−3^ during the flight (8:54–17:30 UTC). Additionally, slight variations were observed in the vertical eBC profile within the first 20 m on this day (Figure 6). These results suggest that the pollutants were well-mixed up to this height.

The vertical distribution of eBC varied with building height and with emission (outdoor) sources and meteorological conditions. At all heights, the highest atmospheric eBC concentrations were observed during the morning rush hour (8:54–11:05 UTC), reaching values between 3.2 and 6.5 µg m^−3^ (Figure 6). Although indoor eBC data measured in the hospital room were not available during the flight period, fortunately the meteorology in the days before and after the flight was similar, especially during the hours corresponding to the flights (TKE = ~1 m^2^ s^−2^; wind direction = southwest; and temperature = ~14 °C). In addition, other pollutants that originated outdoors such as PNC (1.5 × 10^4^ ± 1.4 × 10^3^ cm^−3^), NO (35.8 ± 6.8 µg m^−3^), and NO_2_ (47.3 ± 5.0 µg m^−3^) had comparable concentrations from 16 to 18 February 2021. Considering the above, the pollutant I/O ratios were less than 1, except for that for NO_X_, indicating its outdoor origin. In particular, eBC had an I/O ratio of ~0.95 on 16–18 February 2021.

The vertical distribution of pollutants is more difficult to obtain than the horizontal distribution. Related studies have found a significant correlation between meteorological factors and changes in the NO_2_ I/O ratio at different heights [117]. Rubino et al. [118] measured air pollutants (CO, organic vapors, and PM_10_) between the street level and the top of a tower building (~100 m a.g.l.) and observed a decrease in pollutant levels with increasing height. Yurdakul et al. [119] made similar observations for SO_2_ and NO_2_ in a high-rise building on the campus of the Middle East Technical University in Ankara (Turkey) in both indoor and outdoor air. Differences in PM_x_, ultrafine particles, BC, and particle-bound polycyclic aromatic hydrocarbons (p-PAHs) between night and day were found at different heights of a high-rise apartment building at Feng Chia University (Taiwan) [120]. Since indoor air quality in densely populated urban areas with high-rise buildings is an increasingly serious problem influenced by outdoor sources and environmental conditions, the vertical distribution patterns of traffic-related pollutants must also be examined.

## 4. Conclusions

Both nonregulated (PM_1_, particle number concentration (PNC) such as equivalent black carbon (eBC) including its vertical distribution) and regulated pollutants (PM_10_, PM_2.5_, NO, NO_2_, NO_x_, and O_3_) were measured in real time during two intensive observation periods (summer 2020 and winter 2021) in a sensitive public building (a hospital) in Madrid. The aim of this study is to investigate the relationship between indoor and outdoor pollutants in terms of sources and environmental (outdoor) conditions, providing information on a building type that has been understudied in the literature, as well as on pollutants that have received limited attention due to a lack of regulation.

Indoor particulate matter and related pollutants were influenced by both outdoor sources and indoor activities. PM_10_ concentrations were mainly affected by activities (walking) within the hospital room. In contrast, PNC, eBC, and NO_X_ were influenced by outdoor sources (traffic emissions) via infiltration or natural ventilation through open windows. Different behaviors were observed between particulate matter (PM_x_, PNC, and eBC) and gaseous pollutants (NO_x_ and O_3_).

For particles, infiltration was mainly determined by diffusion capacity, which increased with smaller particle size. Thus, PM_1_ had higher I/O ratios than PM_2.5_ and PM_10_ in both (winter and summer) measurement periods. These small-sized particles are the most harmful to human health, even at relatively low concentrations. eBC, the main component of the smallest particles and a Category I Carcinogen according to the WHO, had higher I/O ratios than PNC (~0.7 in the winter and summer campaigns). Note that outdoor concentrations exceeded 10,000 particles cm^−3^ (the hourly value recommended by the WHO), especially in winter, while indoor concentrations were lower. This could be due to a greater infiltration capacity and a lower removal rate from the indoor environment.

Several drone flights measured the vertical distribution of eBC, which peaked (3.2–6.5 µg m^−3^) during rush hours and had relatively homogeneous concentrations within the height of 20 m a.g.l. The I/O ratios of the gaseous pollutants varied more than those of the particulate pollutants between the measurement periods, with I/O ratios of about 1 in winter for NO_x_. Poor indoor ventilation likely led to NOx accumulation, particularly at night.

Indoor coarse PM_x_ (PM_10_ and PM_2.5_) was mainly generated by the resuspension of particles caused by walking, with 10 min I/O ratios up to 9. The longer occupants stayed in the hospital room, the higher the indoor PM_x_ levels. In contrast, the opening of windows allowed outdoor air to freely move into the indoor environment, increasing the I/O ratios, especially for traffic-related pollutants. Ratios close to 1 (or higher) were found for PNC (up to 2.7) and eBC and NO_x_ (up to 4.0). A synergistic effect was observed between the open window and people in the hospital room. I/O ratios were increased for both PM_x_ (due to resuspension) and traffic-related pollutants (due to outdoor air infiltration). After several hours, indoor concentrations tend to return to previous I/O values.

Meteorology, specifically atmospheric stability/turbulence conditions, strongly influenced the I/O ratios in both occupied and non-occupied conditions. The highest turbulence values (Turbulent Kinetic Energy, TKE > 1 m^2^ s^−^^2^) resulted in rapid outdoor concentration changes with slower indoor response times, with the highest pollutant I/O ratios found under these atmospheric dispersive conditions. These differences were statistically significant (*p*-value < 0.05), except for NO_2_ in the summer, possibly due to its secondary nature. In addition, the pollutant I/O ratios decreased with winds from the west (from the room window) and east (from traffic roads) directions during unstable conditions in both the summer and winter campaigns.

Undoubtedly, more emphasis needs to be placed on nonregulated pollutants, especially those considered to be potential toxicants, as a basis for epidemiological studies. The results of this study will therefore be useful for preventing, controlling, and mitigating the effects of environmental (outdoor and indoor) pollution on indoor air quality in buildings with similar characteristics. Effective ways to reduce the impact of outdoor pollutants and indoor activities on indoor air quality include measures such as ventilation by opening windows during periods of instability, vacuum cleaning to prevent the resuspension of particles, and air purification systems. Furthermore, this information underscores the importance of assessing the influence of outdoor particulate matter and related pollutants on indoor air quality under different scenarios, sources, and meteorology using ambient fixed-site monitoring. In this regard, further research is needed to provide accurate estimates for the development of air quality regulations to promote a healthy indoor environments. The AIRTEC2 project (Evaluación integral de la calidad del aire urbano y cambio climático—2) will continue investigating the mechanisms of I-O transport. This research is essential for establishing preventive measures and improving indoor air quality.

## Figures and Tables

**Table 1:** Sampling points, measurements, and instruments during hospital field campaigns. In this table, “IN” refers to the indoor measurements and “OUT” to the outdoor ones.

Sampling Point	Parameters	Instrument (IN/OUT)	Measurement Size Range (µm)	Time Resolution	Data Coverage (%)
Summer field campaign (23 June to 11 July 2020)					
Room	PNC	CPC, TSI model 3772 (IN) CPC, Aerosol Dynamics Inc. model MAGIC™ (OUT)	>0.01 0.005 to 2.5	1 min 1 s	
	eBC	2 MicroAeth^®^ model AE51 (IN + OUT)	Total	1 min	
	PM_10_, PM_2.5_, PM_1_	Grimm model EDM365 (IN)	Mass concentration of particles < 10, <2.5, and <1 µm respectively	1 min	
	NO, NO_2_, NO_x_	NO_x_ analyzer, Thermo Sci. model 42i (IN) NO_x_ analyzer, Thermo Sci. model 42i-TL (OUT) Palmes-type passive diffusion tubes (IN and OUT)	0–500 ppb	1 min	
CIEMAT	PM_10_, PM_2.5_	TEOM^®^ 1405-DF (OUT)	Particles < 10 and <2.5 µm, respectively	5 min	
Hospital building roof (~34 m a.g.l.)	T, RH, WS, WD, TKE, u*, SH	Portable meteorological mast		10 min	
Winter field campaign (9–28 February 2021)					
Room	PNC	CPC, TSI model 3772 (IN) CPC, Aerosol Dynamics Inc. model MAGIC™ (OUT)	>0.01 0.005 to 2.5	1 min 1 s	
	eBC	MicroAeth^®^ model MA200 (IN) MicroAeth^®^ model AE51 (OUT) ^(1)^	Total	1 min	
	PM_10_, PM_2.5_, PM_1_	Grimm model EDM365 (IN) Grimm model 11D (OUT)	Particles < 10, <2.5, and <1 µm, respectively	1 min	
	NO, NO_2_, NO_x_	NO_x_ analyzer, Thermo Sci. model 42i (IN) NO_x_ analyzer, Thermo Sci. model 42i-TL (OUT) Palmes-type passive diffusion tubes (IN and OUT)	0–500 ppb	1 min	
	O_3_	Ozone Analyzer, Thermo Environmental Instruments model 49 (IN) Ozone Analyzer, Sabio model 6030 (OUT)	0–500 ppb	1 min	
Hospital building roof (~34 m a.g.l.)	T, RH, WS, WD, TKE, u*, SH	Portable meteorological mast		10 min	

^(1)^ Vertical profiles of eBC collected by drone equipped with microAeth^®^ AE51.

**Table 2:** The geometric mean I/O ratios of pollutants (PNC, PM_10_, PM_2.5_, PM_1_, eBC, NO, NO_2_, and O_3_) measured in the hospital room during the summer (23 June to 11 July 2020) and winter (9–28 February 2021) campaigns in the absence of indoor activities, considering the periods (1 and 2) in which the passive diffusion tubes were installed. The I/O ratios for NO_2_ measured by the passive diffusion tube are included in this table. TKE was included as a parameter indicative of micro-scale turbulent conditions. The geometric mean of the hourly ratios was used to calculate the mean I/O ratio (I/O rat.) and 95% confidence intervals (95% CIs in parentheses) of the pollutants measured in this study.

	TKE (m^2^ s^−2^ )	PNC I/O rat. (95% CI)	PM_10_ I/O rat. (95% CI)	PM_2.5_ I/O rat. (95% CI)	PM_1_ I/O rat. (95% CI)	eBC I/O rat. (95% CI)	NO I/O rat. (95% CI)	NO_2_ I/O rat. (95% CI)	NO_2_ Passive Tubes	O_3_ I/O rat. (95% CI)
Summer campaign 2020										
All periods	1.7	0.78 (0.77,0.79)	0.17 (0.16,0.17)	0.22 (0.21,0.22)	-	0.76 (0.75,0.77)	0.35 (0.34,0.37)	1.05 (1.03,1.08)	-	-
Period 1 (June 23 (00:00 UTC) – July 7 (07:40 UTC))	1.5	0.64 (0.62,0.66)	0.14 (0.14,0.15)	0.21 (0.21,0.22)	-	0.71 (0.69,0.73)	0.29 (0.26,0.32)	0.89 (0.85,0.92)	0.8	-
Period 2 (July 7 (07:50 UTC) – 11 (23:50 UTC))^1^	2.5	0.80 (0.78,0.82)	0.13 (0.12,0.13)	0.16 (0.16,0.17)	-	0.68 (0.66,0.70)	0.44 (0.41,0.48)	1.21 (1.17,1.27)	-	-
Winter campaign 2021										
All periods	1.0	0.58 (0.57,0.59)	0.25 (0.24,0.25)	0.30 (0.29,0.30)	0.42 (0.41,0.42)	0.78 (0.76,0.79)	0.95 (0.92,0.98)	0.95 (0.93,0.97)	-	0.43 (0.41,0.44)
Period 1 (February 9 (00:00 UTC) – 15 (10:40 UTC))	1.0	0.53 (0.52,0.54)	0.22 (0.21,0.22)	0.27 (0.27,0.28)	0.38 (0.38,0.39)	0.73 (0.71,0.74)	1.06 (1.02,1.10)	0.92 (0.90,0.94)	1.0	0.43 (0.41,0.44)
Period 2 (February 15 (10:50 UTC) – 28 (23:50 UTC)) ^1^	1.0	0.48 (0.47,0.49)	0.18 (0.17,1.19)	0.26 (0.25,0.26)	0.38 (0.37,0.39)	0.68 (0.64,0.71)	0.99 (0.92,1.07)	0.69 (0.67,0.71)	-	0.60 (0.56,0.64)

^1^ Passive values in period 2 are not considered in this table because they include occupancy periods.

## Data Availability

Data are available through the authors.

## Supplementary Materials

The following supporting information can be downloaded at https://www.mdpi.com/article/10.3390/ijerph22081175/s1, Figure S1: Parameters measured Indoor (In.) and Outdoor (Out.) at the ETSII classroom during the summer campaign in June–July 2020: PNC (Ultrafine Particle Number Concentration in cm^–3^), PM_10_, PM_2.5_ and PM_1_ (in μg·cm^–3^), eBC (equivalent Black Carbon in μg·cm^–3^) and NO and NO_2_ (in μg·cm^–3^). WS (Wind Speed in m s^–1^) and WD (Wind Direction in degrees) values have been included as a proxy for the ventilation atmospheric conditions during the campaign. Out. refers to outdoor measurements and are represented by a solid area on the left vertical axis, while In. refers to indoor measurements and is represented by a line on the right vertical axis. Different situations such as Saharan dust, presence in the classroom (instrument maintenance and data backup) or other type of sampling (biological sampling) and window opening are indicated in the figure; Figure S2: Bivariate polar plots of PNC (Ultrafine Particle Number Concentration in cm^–3^), PM_10_, PM_2.5_ and PM_1_ (in μg·cm^–3^), eBC (equivalent Black Carbon in μg·cm^–3^) and NO, NO_2_ and O_3_ (in μg·cm^–3^) in all sites during the summer (June–July 2020) and winter (February 2021) field campaigns; Figure S3: Parameters measured Indoor (In.) and Outdoor (Out.) at the ETSII classroom during the winter campaign in February 2021: PNC (Ultrafine Particle Number Concentration in cm^–3^), PM_10_, PM_2.5_ and PM_1_ (in μg·cm^–3^), eBC (equivalent Black Carbon in μg·cm^–3^) and NO, NO_2_ and O_3_ (in μg·cm^–3^). WS (Wind Speed in m s^–1^) and WD (Wind Direction in degrees) values have been included as a proxy for the ventilation atmospheric conditions during the campaign. Out. refers to outdoor measurements and is represented by a solid area on the left vertical axis, while In. refers to indoor measurements and is represented by a line on the right vertical axis. Different situations such as Saharan dust, presence in the classroom (instrument maintenance and data backup) or other type of sampling (biological sampling) and window opening are indicated in the figure; Figure S4: Sensitivity analysis of I/O pollutant ratios to TKE for summer (2020) and winter (2021) campaigns; Table S1: Data coverage for each pollutant during the field campaigns: summer (June 23 to 11 July 2020) and winter (9–28 February 2021); Table S2: Arithmetic means and range [min-max] of one-hour data of pollutants (PNC, eBC, PM_10_, PM_2.5_, PM_1_, NO, NO_2_, and O_3_) measured in the hospital room during the summer (23 June to 11 July 2020) and winter (9–28 February 2021) campaigns. All units are in µg m^−3^ except for PNC (particle number concentration × 10_3_ in cm^–3^); Table S3: T-student results for the difference of indoor/outdoor (I/O) ratios for all pollutants (PNC, PM_10_, PM_2.5_, PM_1_, eBC, NO, NO_2_ and O_3_) measured in the hospital room during the non-occupancy and the atmospheric stability using the TKE parameter as an indicator of the atmospheric diffusion (values > 1 m^2^ s^−2^, unstable conditions and ≤1 m^2^ s^−2^, stable conditions). Bold type indicates non-significant results (*p* > 0.05); Table S4: Summary of the linear regression model for each pollutant measured in each campaign (summer 2020 and winter 2021), where the dependent variable is the logarithm of the ratio and the independent variables are the stability factor (TKE > 1 m^2^ s^−2^) and the wind direction factor taking into account the eight directional sectors (N, NE, E, SE, S, SW, W and NW). The reliability of the model is assessed by the p-value at a significance level of 0.05 and the variability explained by the adjusted R2.
