# Supplementary material for: Indoor/Outdoor Particulate Matter and Related Pollutants in a Sensitive Public Building in Madrid (Spain)
Source: Int J Environ Res Public Health. 2025 Jul 25;22(8):1175. doi: 10.3390/ijerph22081175 (PMC12385876; doi:10.3390/ijerph22081175)
Supplement: Supplementary file 1 [file ijerph-22-01175-s001.zip › ijerph-3654448-supplementary.pdf]

SUPPLEMENTARY DATA

Indoor/outdoor particulate matter and related pollutants in a sensitive public building in Madrid (Spain)

Elisabeth Alonso-Blanco [1,*], Francisco Javier Gómez-Moreno [1], Elías Díaz-Ramiro [1], Javier Fernández [1],
Esther Coz [1], Carlos Yagüe [2], Carlos Román-Cascón [3], Dulcenombre Gómez-Garre [4], Adolfo Narros [5], Rafael Borge [5] and Begoña Artíñano [1]

[1] Department of Environment, Center for Energy, Environmental and Technological Research (CIEMAT), Avenida Complutense 40, 28040 Madrid, Spain; fj.gomez@ciemat.es (F.J.G.-M.); elias.diaz@ciemat.es (E.D.-R.); javier.fernandezg@ciemat.es (J.F.); esther.coz@ciemat.es (E.C.); b.artinano@ciemat.es (B.A.)
[2] Departamento de Física de la Tierra y Astrofísica, Universidad Complutense de Madrid, 28040 Madrid, Spain; carlos@ucm.es
[3] Department of Applied Physics, Marine and Environmental Sciences Faculty, INMAR, CEIMAR, University of Cadiz, Puerto Real, 11510 Cádiz, Spain; carlos.roman@uca.es
[4] Laboratorio de Biología Vascular y Microbiota, Hospital Clínico San Carlos; Departamento de Fisiología, Facultad de Medicina, Universidad Complutense de Madrid (UCM); IdISSC, 2nd Floor North, C/Profesor Martín Lagos, s/n, 28040 Madrid, Spain; mgomezgarre@salud.madrid.org
[5] Departamento de Ingeniería Química Industrial y del Medio Ambiente, Universidad Politécnica de Madrid (UPM), 28006 Madrid, Spain; adolfo.narros@upm.es (A.N.); rafael.borge@upm.es (R.B.)
* Correspondence: elisabeth.alonso@ciemat.es

Table S1. Data coverage for each pollutant during the field campaigns: summer (June 23 to July 11, 2020) and winter (February 9-28, 2021).

	Period	Outdoor/Indoor	PNC	PM_{10}	$PM_{2.5}$	PM_1	eBC	NO	NO_2	O_3
Summer (2020) campaign (June 23 to July 11, 2020)	19 days	Outdoor	83%	100%	100%	-	100%	87%	87%	-
		Indoor	82%	100%	100%	100%	100%	100%	100%	-
Winter (2021) campaign (February 9-28, 2021)	20 days	Outdoor	100%	100%	100%	100%	100%	100%	100%	100%
		Indoor	100%	100%	100%	100%	88%	100%	100%	65%

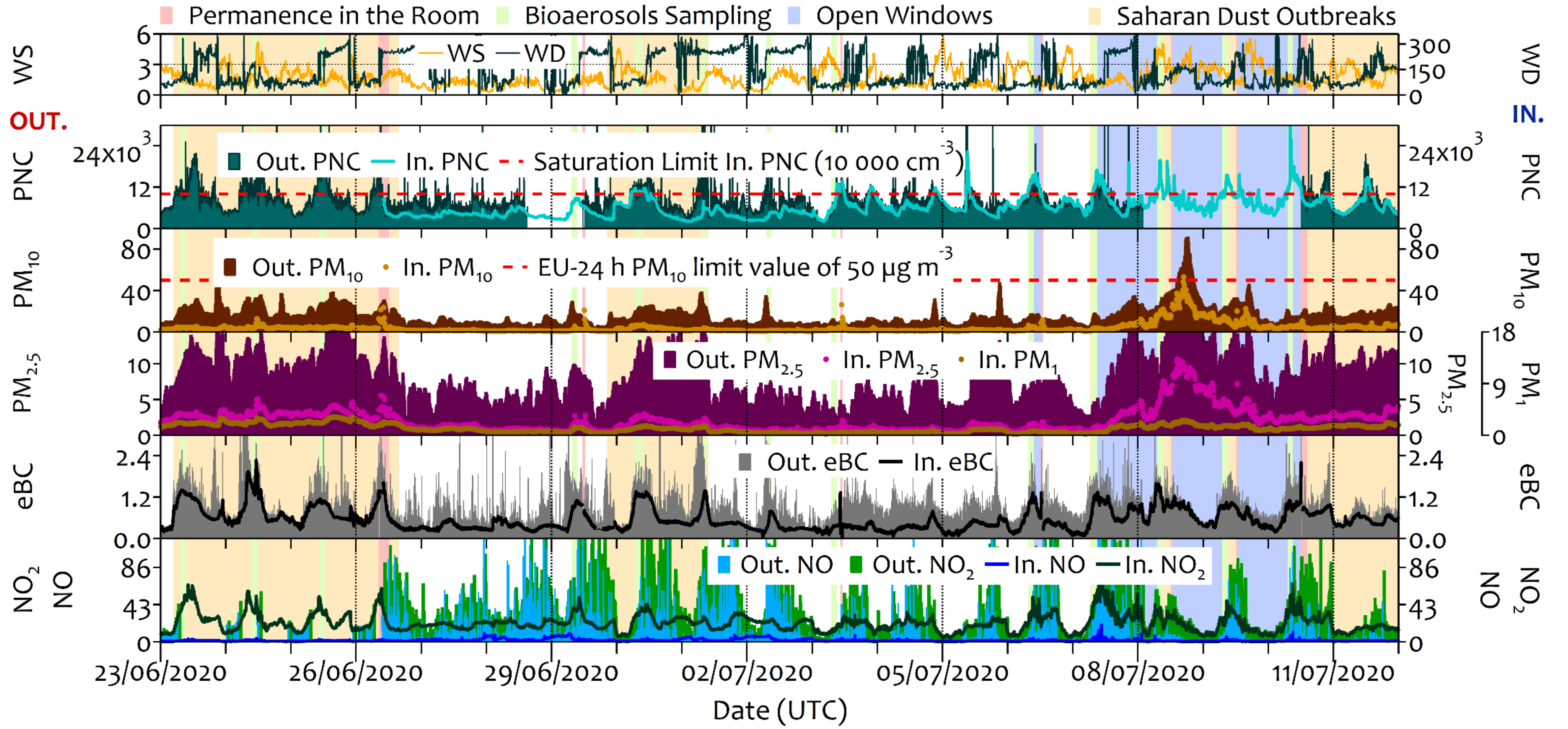

Figure S1. Parameters measured Indoor (In.) and Outdoor (Out.) at the ETSII classroom during the summer campaign in June-July 2020: PNC (Ultrafine Particle Number Concentration in cm^{-3}), PM_{10}, $PM_{2.5}$ and PM_1 (in $\mu g \cdot cm^{-3}$), eBC (equivalent Black Carbon in $\mu g \cdot cm^{-3}$) and NO and NO_2 (in $\mu g \cdot cm^{-3}$). WS (Wind Speed in m s^{-1}) and WD (Wind Direction in degrees) values have been included as a proxy for the ventilation atmospheric conditions during the campaign. Out. refers to outdoor measurements and are represented by a solid area on the left vertical axis, while In. refers to indoor measurements and is represented by a line on the right vertical axis. Different situations such as Saharan dust, presence in the classroom (instrument maintenance and data backup) or other type of sampling (biological sampling) and window opening are indicated in the figure.

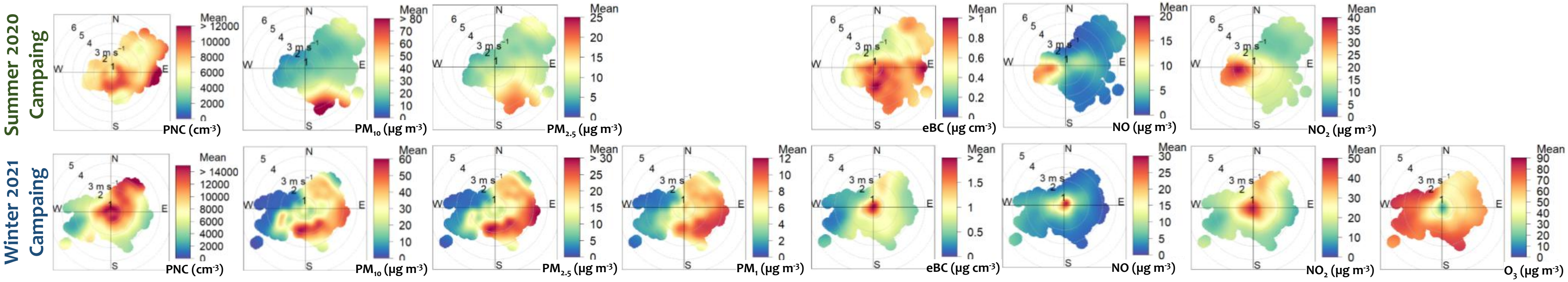

Figure S2. Bivariate polar plots of PNC (Ultrafine Particle Number Concentration in cm^{-3}), PM_{10}, $PM_{2.5}$ and PM_1 (in $\mu g \cdot cm^{-3}$), eBC (equivalent Black Carbon in $\mu g \cdot cm^{-3}$) and NO, NO_2 and O_3 (in $\mu g \cdot cm^{-3}$) in all sites during the summer (June-July 2020) and winter (February 2021) field campaigns.

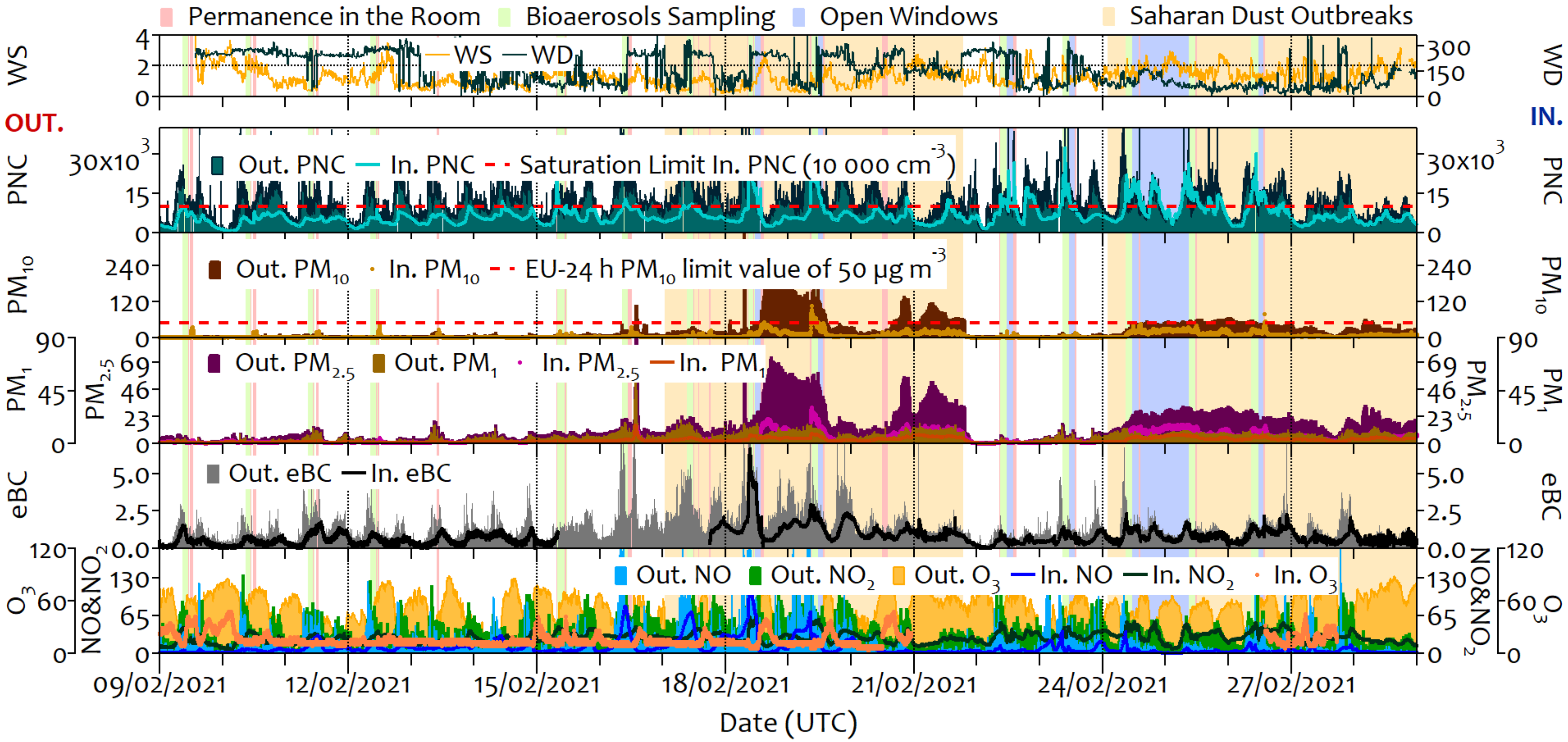

Figure S3. Parameters measured Indoor (In.) and Outdoor (Out.) at the ETSII classroom during the winter campaign in February 2021: PNC (Ultrafine Particle Number Concentration in cm^{-3}), PM_{10}, $PM_{2.5}$ and PM_1 (in µg·cm^{-3}), eBC (equivalent Black Carbon in µg·cm^{-3}) and NO, NO_2 and O_3 (in µg·cm^{-3}). WS (Wind Speed in m s^{-1}) and WD (Wind Direction in degrees) values have been included as a proxy for the ventilation

atmospheric conditions during the campaign. Out. refers to outdoor measurements and is represented by a solid area on the left vertical axis, while In. refers to indoor measurements and is represented by a line on the right vertical axis. Different situations such as Saharan dust, presence in the classroom (instrument maintenance and data backup) or other type of sampling (biological sampling) and window opening are indicated in the figure.

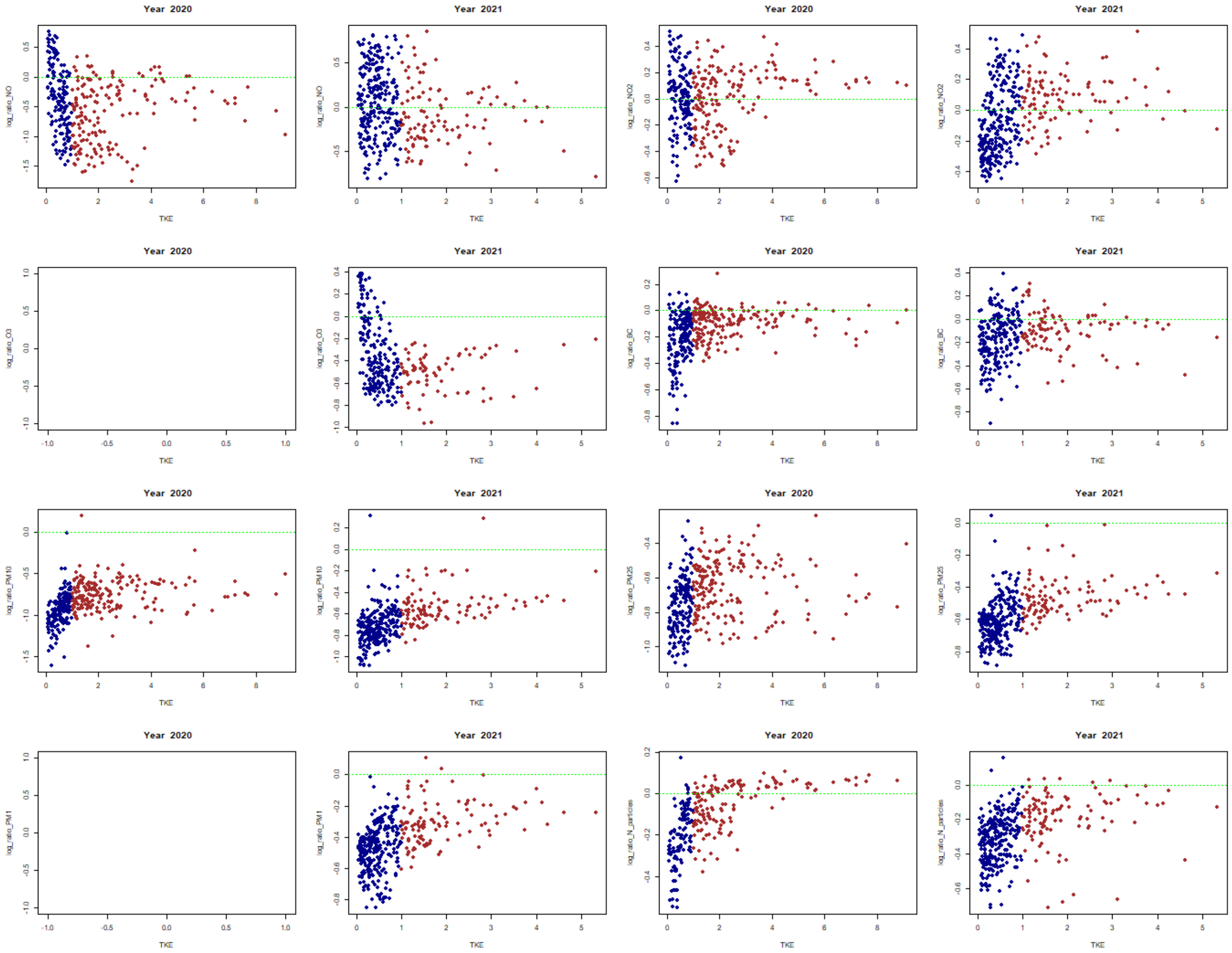

Figure S4. Sensitivity analysis of I/O pollutant ratios to TKE for summer (2020) and winter (2021) campaigns.

Table S2 Arithmetic means and range [min-max] of one-hour data of pollutants (PNC, eBC, PM_{10}, $PM_{2.5}$, PM_1, NO, NO_2, and O_3) measured in the hospital room during the summer (June 23 to July 11, 2020) and winter (February 9-28, 2021) campaigns. All units are in μg m^{-3} except for PNC (particle number concentration × 10^3 in cm^{-3}).

		**PNC**	**PM_{10}**	**$PM_{2.5}$**	**PM_1**	**eBC**	**NO**	**NO_2**	**NO_2 passive tubes**	**O_3**
Summer campaign 2020										
Indoor	All period	6.3 [2.0, 31.4]	3.9 [0.4, 53.0]	2.1 [0.4, 10.7]	1.2 [0.2, 3.2]	0.6 [0.0, 2.2]	1.6 [0.1, 13.1]	21.9 [4.9, 75.9]		-
Indoor	Period 1 (June 23 (00:00 UTC) –July 7 (7:40 UTC))	4.6 [2.0, 11.5]	2.8 [0.5, 52.4]	2.0 [0.5, 5.6]	1.5 [0.4, 3.2]	0.6 [0.1, 2.2]	1.9 [0.3, 9.2]	23.2 [6.0, 61.9]	21.7	-
Indoor	Period 2 (July 7 (7:50 UTC)–9 (7:30 UTC))	6.7 [2.1, 21.0]	4.6 [0.4, 53.0]	1.9 [0.4, 10.7]	0.8 [0.2, 2.6]	0.5 [0.0, 1.6]	1.5 [0.1, 13.1]	21.2 [4.9, 75.9]	25.8	-
Outdoor	All period	7.7 [1.9, 44.8]	17.2 [1.4, 92.3]	8.3 [0.1, 22.1]	-	0.7 [0.1, 4.7]	7.5 [0.4, 230.9]	23.4 [2.5, 233.3]		-
Outdoor	Period 1 (June 23 (00:00 UTC) –July 7 (7:40 UTC))	8.0 [2.8, 28.7]	17.3 [1.4, 68.2]	8.7 [0.1, 17.3]	-	0.9 [0.2, 4.7]	10.5 [0.8, 230.9]	27.1 [4.0, 233.3]	28.3	-
Outdoor	Period 2 (July 7 (7:50 UTC)–9 (7:30 UTC))	7.5 [1.9, 44.8]	16.3 [5.3, 92.3]	7.4 [1.1, 22.1]	-	0.6 [0.1, 2.4]	6.7 [0.5, 80.8]	22.2 [2.5, 99.6]	22.2	-
Winter campaign 2021										
Indoor	All period	6.7 [0.6, 30.0]	7.1 [0.2, 90.7]	4.4 [0.1, 29.5]	2.7 [0.1, 15.6]	0.8 [0.0, 6.0]	9.8 [0.4, 139.6]	27.4 [6.5, 60.9]		15.6 [5.6, 46.4]
Indoor	Period 1 (February 9 (00:00 UTC)–15 (10:40 UTC))	5.1 [0.6, 16.0]	1.9 [0.4, 32.4]	1.3 [0.4, 4.0]	1.1 [0.3, 2.2]	0.5 [0.0, 1.6]	8.2 [1.2, 30.2]	23.3 [6.5, 45.4]	25.0	16.2 [8.7, 46.4]
Indoor	Period 2 (February 15 (10:50 UTC)–22 (11:20 UTC))	6.8 [1.5, 30.0]	9.5 [0.2, 90.7]	5.9 [0.1, 29.5]	3.5 [0.1, 15.6]	1.2 [0.0, 6.0]	15.7 [1.6, 139.6]	28.7 [12.1, 60.9]	32.5	14.5 [5.6, 44.5]
Outd	All period	11.6 [0.8, 47.3]	28.1 [0.2, 202.5]	14.6 [0.2, 99.1]	6.3 [0.1, 76.5]	1.2 [0.0, 11.1]	12.7 [0.6, 207.8]	32.6 [1.9, 95.7]		46.6 [4.2, 92.6]

Period 1 (February 9 (00:00 UTC)–15 (10:40 UTC))	10.4 [0.8, 31.8]	6.5 [0.9, 16.3]	5.0 [0.8, 13.7]	3.2 [0.5, 10.9]	0.8 [0.0, 3.4]	9.5 [0.6, 88.5]	28.0 [1.9, 91.8]	24.2	49.7 [5.4, 88.3]
Period 2 (February 15 (10:50 UTC)–22 (11:20 UTC))	12.8 [1.0, 47.3]	45.5 [0.2, 202.5]	21.3 [0.2, 99.1]	8.3 [0.1, 76.5]	1.7 [0.0, 11.1]	19.8 [0.8, 207.8]	39.6 [4.0, 95.7]	32.9	39.6 [4.2, 92.6]

Table S3 T-student results for the difference of indoor/outdoor (I/O) ratios for all pollutants (PNC, PM_{10}, $PM_{2.5}$, PM_1, eBC, NO, NO_2 and O_3) measured in the hospital room during the non-occupancy and the atmospheric stability using the TKE parameter as an indicator of the atmospheric diffusion (values > 1 $m^2\ s^{-2}$, unstable conditions and ≤ 1 $m^2\ s^{-2}$, stable conditions). Bold type indicates non-significant results (p > 0.05).

	**t**	**df**	**p-value**
	**Summer campaign 2020**		
PNC	-10.512	227.09	< 2.2e-16
PM_{10}	-9.9664	355.4	< 2.2e-16
$PM_{2.5}$	-6.6943	363.88	8.22E-11
PM_1			
eBC	-7.3067	267.4	3.16e-12
NO	3.8569	308.18	1.40E-04
NO_2	0.3592	315.02	**7.20E-01**
O_3			
	**Winter campaign 2021**		
PNC	-7.3758	192.35	4.75E-12
PM_{10}	-10.26	202.89	< 2.20E-16
$PM_{2.5}$	-10.907	216.3	< 2.2e-16

PM_1	-10.433	222.83	< 2.2e-16
eBC	-3.6273	237.18	3.51E-04
NO	3.3241	227.81	1.03E-03
NO_2	-8.2603	278.22	5.96E-15
O_3	6.0078	231.34	7.24E-09

Table S4 Summary of the linear regression model for each pollutant measured in each campaign (summer 2020 and winter 2021), where the dependent variable is the logarithm of the ratio and the independent variables are the stability factor (TKE > 1 $m^2 s^{-2}$) and the wind direction factor taking into account the eight directional sectors (N, NE, E, SE, S, SW, W and NW). The reliability of the model is assessed by the p-value at a significance level of 0.05 and the variability explained by the adjusted R2.

			**NE**	**E**	**SE**	**S**	**SW**	**W**	**NW**	**Ad R-squared**	**p-value**
**Summer campaign 2020**	PNC	*Estimate*	0.16683	0.14853	0.08622	0.19282	0.12478	0.02041	-0.07096	0.5281	< 2.2e-16
		Std. Error	0.04778	0.04748	0.0528	0.05784	0.05060	0.04692	0.05525		
		t value	3.492	3.128	1.632	3.334	2.466	0.435	-1.284		
		Pr(>|t|)	**0.000565*** **	**0.001964** **	0.103822	**0.000984*** **	**0.014319***	0.663876	0.200193		
	PM_{10}	*Estimate*	0.01315	0.12902	0.01743	0.08803	0.09108	0.01714	-0.09611	0.269	< 2.2e-16
		Std. Error	0.08391	0.08308	0.09114	0.09916	0.08699	0.08311	0.09592		
		t value	0.157	1.553	0.191	0.888	1.047	0.206	-1.002		
		Pr(>|t|)	0.876	0.121	0.848	0.375	0.296	0.837	0.317		
	$PM_{2.5}$	*Estimate*	-1.131e-03	9.441e-02	3.647e-02	5.864e-02	1.359e-02	4.614e-05	-5.528e-02	0.1517	1.42e-11
		Std. Error	7.239e-02	7.167e-02	7.863e-02	8.554e-02	7.505e-02	7.170e-02	8.275e-02		
		t value	-0.016	1.317	0.464	0.686	0.181	0.001	-0.668		
		Pr(>|t|)	0.988	0.189	0.643	0.493	0.856	0.999	0.505		
	PM_1	*Estimate*									

			NE	E	SE	S	SW	W	NW	Ad R-squared	p-value
		Std. Error									
		t value									
		Pr(>|t|)									
	eBC	*Estimate*	-0.08868	-0.02402	-0.07165	-0.02406	-0.02804	-0.09492	-0.12316	0.1463	4.046e-11
		Std. Error	0.06928	0.06860	0.07526	0.08187	0.07183	0.06862	0.07920		
		t value	-1.280	-0.350	-0.952	-0.294	-0.390	-1.383	-1.555		
		Pr(>|t|)	0.2014	0.7264	0.3417	0.7690	0.6965	0.1674	0.1208		
	NO	*Estimate*	-0.54118	-0.85234	-0.49738	-0.31296	-1.06910	-0.92448	-0.32319	0.226088	1.534e-13
		Std. Error	0.22762	0.22556	0.25075	0.27864	0.23867	0.22518	0.26646		
		t value	-2.378	-3.779	-1.984	-1.123	-4.479	-4.106	-1.213		
		Pr(>|t|)	**0.018029***	**0.000189*****	**0.000189*****	0.262226	**1.05e-05*****	**5.15e-05*****	0.226088		
	NO_2	*Estimate*	-0.09421	-0.12514	-0.19890	-0.08327	-0.40055	-0.40196	-0.14897	0.3387	< 2.2e-16
		Std. Error	0.08848	0.08768	0.09747	0.10831	0.09278	0.08753	0.10358		
		t value	-1.065	-1.427	-2.041	-0.769	-4.317	-4.592	-1.438		
		Pr(>|t|)	0.28783	0.15452	**0.04213***	0.44258	2.12e-05	6.36e-06	0.15139		
	O_3	*Estimate*									
		Std. Error									
		t value									
		Pr(>|t|)									
**Winter campaign**	PNC	*Estimate*		-0.02798	-0.04914	-0.05820	-0.00931	-0.11325	-0.07831	0.1794	3.472e-14
		Std. Error		0.02309	0.02769	0.03510	0.02500	0.02337	0.07215		
		t value		-1.212	-1.775	-1.658	-0.372	-4.847	-1.085		
		Pr(>|t|)		0.2265	**0.0768.**	**0.0982.**	0.7099	**1.87e-06*****	**0.2785**		
	PM_{10}	*Estimate*		-0.004428	-0.004428	-0.015229	0.025506	0.035199	0.066925	0.2226	< 2.2e-16
		Std. Error		0.026606	0.026606	0.040444	0.028811	0.026923	0.083137		
		t value		-0.166	-0.166	-0.377	0.885	1.307	0.805		
		Pr(>|t|)		0.868	0.790	0.707	0.377	0.192	0.421		

		NE	E	SE	S	SW	W	NW	Ad R-squared	p-value
$PM_{2.5}$	*Estimate*		-0.011964	0.003274	0.014495	0.028488	0.042034	0.089174	0.2405	< 2.2e-16
	Std. Error		0.020788	0.024929	0.031600	0.022511	0.021036	0.064958		
	t value		-0.576	0.131	0.459	1.266	1.998	1.373		
	Pr(>|t|)		0.5653	0.8956	0.6467	0.2065	**0.0465***	0.1707		
PM_1	*Estimate*		-0.054514	-0.029752	0.001413	0.033983	0.061508	0.169167	0.2793	< 2.2e-16
	Std. Error		0.023076	0.027673	0.035078	0.024988	0.023351	0.072107		
	t value		-2.362	-1.075	0.040	1.360	2.634	2.346		
	Pr(>|t|)		**0.0187***	0.2830	0.9679	0.1747	**0.0088****	**0.0195***		
eBC	*Estimate*		-0.019129	-0.009508	-0.058490	-0.060221	-0.119123	0.105721	0.06757	0.0002015
	Std. Error		0.034194	0.039876	0.051190	0.036921	0.033612	0.098145		
	t value		-0.559	-0.238	-1.143	-1.631	-3.544	1.077		
	Pr(>|t|)		0.576284	0.811704	0.254113	0.103916	**0.000457*****	0.282259		
NO	*Estimate*		0.03290	0.03601	-0.07382	-0.10636	-0.20027	0.19948	0.0734	1.46e-05
	Std. Error		0.05928	0.07109	0.09011	0.06419	0.05999	0.18524		
	t value		0.555	0.507	-0.819	-1.657	-3.339	1.077		
	Pr(>|t|)		0.579207	0.612759	0.413211	**0.098437.**	**0.000931*****	0.282264		
NO_2	*Estimate*		-0.06307	-0.10181	-0.12823	-0.07923	-0.15078	-0.05456	0.1545	5.13e-12
	Std. Error		0.03333	0.03997	0.05066	0.03609	0.03372	0.10414		
	t value		-1.892	-2.547	-2.531	-2.195	-4.471	-0.524		
	Pr(>|t|)		**0.0593.**	**0.0593.**	**0.0118***	**0.0118***	**1.05e-05*****	0.6007		
O_3	*Estimate*		0.12602	-0.02126	-0.21635	-0.01653	-0.08130	-0.31447	0.1318	9.784e-08
	Std. Error		0.06071	0.07280	0.08999	0.06027	0.05837	0.14998		
	t value		2.076	-0.292	-2.404	-0.274	-1.393	-2.097		
	Pr(>|t|)		**0.03885***	0.77045	**0.01688***	0.78413	0.16483	**0.03694***		

Significance codes: *** p-value < 0.001, ** p-value < 0.01, * p-value < 0.05, **.** p-value < 0.1 and “” p-value < 1.
